# Supplementary material for: The relationship between social safeness and pleasure and resilience levels among university athletes: A descriptive study
Source: PLoS One. 2025 Jan 14;20(1):e0315889. doi: 10.1371/journal.pone.0315889 (PMC11731725; doi:10.1371/journal.pone.0315889)
Supplement: S1 File — Details for accessing data through the Data Access Committee. (DOCX) [file pone.0315889.s001.docx]

**The Relationship between Social Safeness and Pleasure and Resilience Levels among University Athletes:** *A Descriptive Study*

^1^Veysel TEMEL, ^2^Nurhan Hümeyra ÖZÇELİK

^1^Karamanoglu Mehmetbey University, Faculty of Sport Sciences, Karaman, [veyseltemel@kmu.edu.tr](mailto:veyseltemel@kmu.edu.tr).

^2^Karamanoglu Mehmetbey University, Faculty of Health Sciences, Karaman, [nurhanhumeyraozcelik@gmail.com](mailto:nurhanhumeyraozcelik@gmail.com).

This study investigates the correlation between social safeness and pleasure and resilience among university students engaged in sports. A total of 350 participants (mean age, 21.09 ± 3.12 years), comprising 239 females and 111 males, were included in the sample.

The data of the study were collected between May and June 2024 with the ethics committee approval permission from Karamanoğlu Mehmetbey University Social and Human Scientific Research Ethics Committee (Date: 22.04.2024, Decision No: 06-2024/131). Informed written consent was obtained from all participants. Ethical rules were followed during the research. Permission to use the “*Social Safeness And Pleasure Scale*” developed by Gilbert et al. and adapted into Turkish by Akın et al. and the “*Resilience Scale*” developed by Wagnild et al. and adapted into Turkish by Terzi was obtained from the first author via e-mail.

The authors whose names are listed certify that they have no conflicts of interest regarding personal or professional relationships, nor any financial interests in this manuscript. The authors confirm their specific contributions to the work presented. The authors are in agreement on the conclusions, implications, or opinions stated in the manuscript reported. All authors give consent to the submission.

The research data will be shared publicly. The data is included in the publication. It will be sent via spss when requested. The data has been organized and anonymized to include all raw data and analysis results obtained as a result of the study.

**Contact Information For A Data Access Committee, Ethics Committee;**

1. Prof. Dr. Murat TEKİN, Karamanoglu Mehmetbey University, Faculty of Sports Sciences, Department Head And Ethics Committee Member, [murattekin76@gmail.com](mailto:murattekin76@gmail.com), +90.338.2262117
2. Prof. Dr. Elif BİROL, Karamanoglu Mehmetbey University, Faculty of Sports Sciences, Department Head And Ethics Committee Member, [elifaydin@kmu.edu.tr](mailto:elifaydin@kmu.edu.tr), +90.338.2264217
3. Prof. Dr. Sefa LÖK, Karamanoglu Mehmetbey University, Faculty of Sports Sciences, Department Head And Ethics Committee Member, sefalok[@gmail.com](mailto:murattekin76@gmail.com), +90.338.2262110

Assoc. Prof. Veysel TEMEL
