# Supplementary material for: The relationship between social safeness and pleasure and resilience levels among university athletes: A descriptive study
Source: PLoS One. 2025 Jan 14;20(1):e0315889. doi: 10.1371/journal.pone.0315889 (PMC11731725; doi:10.1371/journal.pone.0315889)
Supplement: Attachment — Submitted filename: Response to Reviewers.docx [file pone.0315889.s004.docx]

The rebuttal letter

Manuscript Number	: PONE-D-24-27311R1

Manuscript Title	: The Relationship between Social Safeness and Pleasure and Resilience Levels among University Athletes: A Descriptive Study

Dear Academic Editor and Reviewers,

First of all, I would like to thank you for your evaluations, opinions, suggestions and comments. My responses to the evaluations and comments made are written below.

Best regards

Dr. Veysel TEMEL

**Journal Requirements**

1. Please review your reference list to ensure that it is complete and correct. If you have cited papers that have been retracted, please include the rationale for doing so in the manuscript text, or remove these references and replace them with relevant current references. Any changes to the reference list should be mentioned in the rebuttal letter that accompanies your revised manuscript. If you need to cite a retracted article, indicate the article’s retracted status in the References list and also include a citation and full reference for the retraction notice.
1. Answer:

Dear Editor,

We have carefully reviewed and addressed all requested revisions regarding the reference list. Necessary updates were made, and any retracted articles were either replaced or retained with clear justification in the manuscript text. Changes to the reference list are highlighted in yellow within the manuscript.

Thank you for your guidance and consideration.

**Reviewers’ comments and responses**

**Reviewer 1
Reviewer 1:** Although not all of my comments were answered in the reply letter, the manuscript managed to incorporate the suggestions and requests I made in round 1. I therefore consider that the manuscript is in a position to be accepted for publication.

**Author Answer:** Thank you very much for your valuable feedback and support throughout the review process. We appreciate your comments, which have helped improve our manuscript.

**Reviewer 2**

**Reviewer 2:** Regarding my comment number 7 ("It seems to me that the term “sport” is not well-defined. It could help to differentiate it from physical activity and physical exercise"): it is not clear where the authors present this information.

**Author Answer:** Thank you for your valuable feedback and for pointing out the need for a clearer definition of the term "sport." In response to your comment, we have revised the manuscript to include a clear distinction between "sport," "physical activity," and "physical exercise." This clarification has been added in lines 57 to 74 of the revised manuscript, where we elaborate on how "sport" differs from general physical activity and exercise in terms of its organized, competitive nature, as well as its connection to skill, strategy, and formalized competition. We have also included relevant references to support these distinctions.

**Reviewer 2:** Regarding my comment number 10 (‘Lines 94-95: How can a study conducting correlations determine interventions that could improve the examined variables?’): I still have the same doubt, and I can't find in the document where this information was added. I would ask the authors to identify the lines.

**Author Answer:** Thank you for your comment regarding the role of correlation analysis in determining interventions. We have revised the manuscript to clarify that correlation analysis, such as the Pearson Product Moment Correlation Analysis, identifies relationships between variables but does not establish causality or direct intervention strategies. We have expanded on this point in the revised manuscript to emphasize that while the observed correlations between social safeness, pleasure, and resilience may suggest potential areas for intervention, further experimental research is needed to confirm specific interventions. We hope this revision adequately addresses your concern. Thank you for your insightful feedback.

**Reviewer 2:** The authors should clarify the inclusion and exclusion criteria, given that not all athletes are involved in the same sports or practice intensities.

**Author Answer:** Following your comment, we have clarified the categorization of participants based on their frequency of sports participation. The participants were divided into the categories of "Seldom," "Sometimes," "Usually," "Often," and "Always," and they are active individuals who engage in sports at sports clubs or sports centers. We have added this clarification to the manuscript.

**Reviewer 2:** There is no p-value =.000

**Author Answer:** Thank you for your comment. We have corrected the p-value notation in the manuscript. The previously mentioned p-value of 0.000 has been revised to p < 0.001, in accordance with statistical reporting standards.

**Reviewer 2:** The analysis of gender differences was introduced in the results, but this aspect could have been mentioned earlier in the introduction to give context to the choice of variables.

**Author Answer:** Thank you for your valuable suggestion. In response to your comment, we have added a discussion of gender differences in the introduction, specifically between lines 117-125. This addition clarifies the rationale for including gender as a variable in the study, providing context for the choice of variables and their relevance to the research.

**Reviewer 2:** The discussion is carried out in a very superficial way, without much reflection and specific practical recommendations for educational institutions to promote well-being and resilience among students.

**Author Answer:** "The discussion section has been revised to provide a more detailed and comprehensive analysis. The changes have been incorporated in lines 325-380, 407-441, 472-534 and 537-565 and are highlighted in yellow for easy reference."

**Reviewer 2:** The statistical analysis revealed significant differences in the resilience subscales between genders, with higher scores for men. This point could be discussed in greater depth, exploring potential reasons for these differences.

**Author Answer:** "The discussion section has been revised to provide a more detailed and comprehensive analysis. The changes have been incorporated in lines 325-352 are highlighted in yellow for easy reference." Also, Thank you for your valuable feedback. We have carefully considered your suggestions and included a comprehensive "Recommendations for Future Research" section in the manuscript, addressing potential avenues for expanding upon the study's findings. We believe these additions will enhance the clarity and relevance of our research within the field.
